# Efficacy of a referral center for patient-centered care in multiple myeloma: a cohort study

**DOI:** 10.1186/s12913-015-1123-6

**Published:** 2015-10-05

**Authors:** Indara C. Saccilotto, Rosane Isabel Bittencourt, Camila C. Fischer, Amanda Quevedo, Vania N. Hirakata, Paulo D. Picon

**Affiliations:** Hospital de Clínicas de Porto Alegre, Rua Ramiro Barcelos, 2350, Santa Cecília, Porto Alegre, RS 90035-903 Brazil; Universidade Federal do Rio Grande do Sul, Avenida Paulo Gama, 110, Farroupilha, Porto Alegre, RS 90040-060 Brazil

**Keywords:** Patient-centered care center, Multiple myeloma, Quality of life, Thalidomide

## Abstract

**Background:**

Within the Brazilian Unified Health System (SUS), Referral Centers (RCs) are care facilities that provide specialized services. The objective of this study was to evaluate the efficacy of care provided to patients with multiple myeloma (MM) at a specialized RC (Hospital de Clínicas de Porto Alegre Referral Center for Multiple Myeloma, CRMM-HCPA) and to compare quality of life between patients with MM treated at CRMM-HCPA and those treated at non-RC facilities.

**Methods:**

A 6-month cohort study was conducted in patients with MM receiving thalidomide from the Rio Grande do Sul State Health Department and treated at CRMM-HCPA and patients receiving treatment at other, non-RC care facilities. Thirty-two patients were included in the study, 19 from CRMM-HCPA and 13 from other institutions. To analyze the efficacy of care provided at CRMM-HCPA, the main outcome measure was the time from diagnosis to referral for autologous hematopoietic stem cell transplantation (HSCT). This outcome measure was assessed using questionnaires specifically designed for this study. Quality of life was also assessed, using the SF-36 questionnaire.

**Results:**

Time from MM diagnosis to referral for autologous HSCT in each group was measured only in patients aged ≤ 65 years (*n* = 25); of these, 15 were recruited from CRMM-HCPA and 10 from other institutions. In this analysis, there was a significant difference (*p* = 0.036) in time elapsed between diagnosis and referral for autologous HSCT, which was significantly shorter for patients treated at CRMM-HCPA (median, 9 months; IQR, 8.5–14.5) than for those treated elsewhere (median, 24 months; IQR, 16–24). On quality of life analysis, there was a significant difference in the Social Functioning domain of the SF-36 questionnaire, which relates to performance of social activities (*p* = 0.02).

**Conclusions:**

The Referral Center model provided seems to be a more efficient treatment strategy as compared with other health care facilities, as it enabled a reduction in time to transplantation. Patients treated at CRMM-HCPA demonstrated greater ease in performing social activities, with less interference from physical or emotional problems.

## Background

Within the Brazilian Unified Health System (Sistema Único de Saúde, SUS), Referral Centers (RCs) are specialized facilities that provide care as part of an innovative partnership between academia and SUS managers. The main objectives of a RC are to provide multidisciplinary care and follow-up, facilitate access to specialized medicines provided by the State Health Departments (SHDs), and enable creation of care quality indicators to improve public health management.

In many conditions, to ensure recovery, enable proper dose adjustment of pharmaceutical therapy, reduce wastefulness, and prevent further suffering to patients, it is essential that the management strategy involve continuous care, guaranteed access to medicines, and close monitoring of the positive and negative effects of treatment [1].

RCs should implement all guidance advocated in the Clinical Protocols and Practice Guidelines published by the Brazilian Ministry of Health (MoH) [1, 2]. With their experience in providing short- and long-term specialty care and follow-up, RCs have become an environment conducive to academic and research activities to better serve the SUS. Furthermore, they have enabled the performance of clinical efficacy research and clinical trials in areas relevant to the SUS.

Through the RC framework, the SHDs – which dispense to the population all medicines provided by the MoH Specialized Component of Pharmaceutical Assistance (*Programa do Componente Especializado*) – can achieve greater control of the volume of medicines required.

At Hospital de Clínicas de Porto Alegre (HCPA), a tertiary referral center and major teaching hospital in Porto Alegre, state of Rio Grande do Sul, Brazil, the operation of a RC is based on the provision of care, in accordance with the treatment routine necessary for treatment of the condition, by a multidisciplinary team of professionals from both the HCPA and the SHD, as well as by undergraduate and graduate students from institutions of higher learning. The expected result of this cooperation is improved access to medicines, treatment adherence, and user satisfaction, as well as savings brought on by improved pharmaceutical management, benefiting patients and the two public institutions alike.

RCs also enable the compilation of specific databases and registries, which can be used in the conduction of clinical efficacy research and for information management purposes, further strengthening technical cooperation among public health institutions and making RCs a very important tool in supporting decision-making and public policymaking by the MoH and, more directly, by SHDs.

Multiple myeloma (MM) is a B-cell neoplasm that affects immunoglobulin (antibody)-producing plasma cells. It is characterized by plasma-cell infiltration of the bone marrow, anchored by endothelial growth factors, and is associated with the action of adhesion molecules, interleukin (IL)-6, IL-3, IL-1 beta, and IL-10, granulocyte- and monocyte-stimulating factors, and tumor necrosis factor alpha.

MM is incurable [3, 4], and may be treated with chemotherapy and new treatment modalities such as proteasome inhibitors and/or immunomodulators, including thalidomide. In the late 1990s, the first reports of thalidomide in the treatment of MM changed the entire panorama of the disease, and VAD (vincristine, doxorubicin [Adriamycin], and dexamethasone) chemotherapy fell into disuse [5].

The choice of anti-myeloma therapy scheme depends on factors such as age, performance status, and presence of comorbidities. Patients younger than 65 years, with performance status 0, 1, or 2, and no severe comorbidities (other neoplasm, grade III–IV heart failure, AIDS with CD4 counts < 200) are candidates for autologous hematopoietic stem cell transplantation (autologous HSCT) immediately after induction therapy [6–9]. Autologous HSCT is the gold-standard treatment for patients under the age of 70, and is known to produce complete remission lasting many years [8, 10, 11]. Delays in treatment initiation have a negative impact on patient response and can delay escalation of therapy to other stages, such as HSCT.

The HCPA Multiple Myeloma Referral Center (*Centro de Referência de Mieloma Múltiplo, CRMM-HCPA*) was established in 2010 to eliminate obstacles to access to SHD-dispensed thalidomide and reduce delays between diagnosis and start of treatment for patients with MM. All MM patients treated via the SUS receive thalidomide in accordance with the National Guidelines for MM, but time to treatment access varies between the different institutions that provide services to SUS.

Taking into account that the services provided by a RC may be considered a new health technology, the present study sought to assess the efficacy of these services within the SUS framework. The main outcome measure was time elapsed between diagnosis of MM and referral for autologous HSCT.

## Methods

We conducted an efficacy analysis of the care provided at CRMM nested in a 6-month ambispective cohort of patients with MM who were receiving thalidomide treatment at CRMM-HCPA, from May 2012 to May 2014. All study procedures were approved by the Research Ethics Committee at Hospital de Clínicas de Porto Alegre (no. 283.728). The comparator group was a sample of patients with MM who were being treated at other facilities. The main outcome measure was the time between diagnosis of MM and referral for HSCT. We also compared quality of life parameters and health service costs between the two groups.

### Sample

The study sample comprised 32 participants with MM who were on thalidomide therapy, all of whom were referred for transplantation: 19 treated at CRMM-HCPA and 13 treated at other health care facilities in the state (control group). All were registered with the SHD and provided written informed consent for participation.

### Procedures

**Fig. 1:**
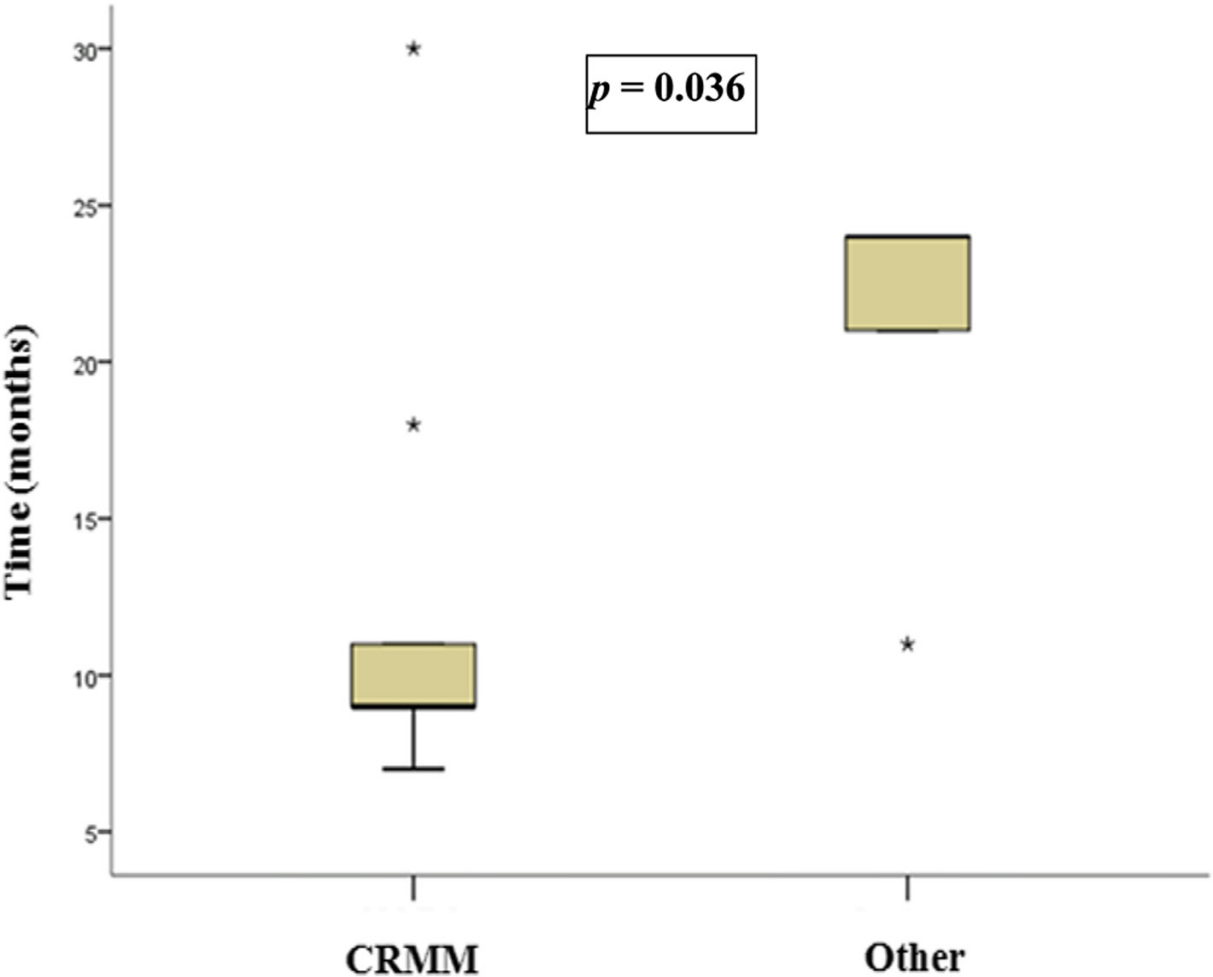
Time from diagnosis to referral for transplantation

Patients from CRMM-HCPA were identified through the records of the hospital’s outpatient MM clinic, where they receive routine care through the SUS. Patients from other institutions were identified from the RS SHD Registry, which was accessed through the Department’s own electronic system. This Registry contains personal information for the patients, such as addresses and telephone numbers, and is used to control dispensation of medicines supplied by the SHD. All participants of this study were treated through the SUS. In the city of Porto Alegre, these patients are referred for treatment at public hospitals after attending municipal-run health clinics; the destination hospital depends on the patient’s home address (Fig. 1).

The quality of life instrument (SF-36 v.2) and a health resources utilization questionnaire were administered to all participants at inclusion (I1) and at the 6-month study visit (I2). At I1, participants also completed a questionnaire designed to collect demographic and treatment data. The SF-36 v.2 was completed without investigator interference. Scores were calculated for analysis of physical, mental, and social functioning.

The health resources utilization questionnaire was employed for comparative analysis of the costs to the SUS of patients treated at the CRMM versus those of patients treated in other facilities.

All hospital cost analyses were conducted from a SUS perspective and all costs were drawn from the SUS master table, as reported by the HCPA billing sector.

### Statistical analysis

For SF-36 v.2 calculation, items were transformed into domains. A different calculation formula is used for each domain. Scores range from 0 to 100, and were calculated with the Health Outcomes Scoring Software 4.5.

In quality of life assessment, descriptive statistical methods were used to obtain measures such as means, standard deviations, and standard errors.

All statistical analyses were carried out in PASW Statistics 18 for Windows. The significance level was set at *p* < 0.05.

The remaining variables of interest were: sex (male or female); age at diagnosis; educational attainment; and quality of life. Fisher’s exact test was used for analysis of sex and educational attainment data, whereas Student’s t-test was used for analysis of age at diagnosis (mean ± standard deviation). The Mann–Whitney U test was used for the variable duration of treatment (median and IQR) in months, and for analysis of time elapsed between diagnosis and referral for HSCT.

For within-group analysis of *P*-values for cost and quality of life data, we conducted a comparative analysis by means of the generalized linear models method with Bonferroni multiple comparisons.

## Results

Quality of life analysis (Table 1) revealed a significant between-group difference in the Social Role Functioning domain, which pertains to involvement in social activities (*p* = 0.02), in favor of the CRMM group.

**Table 1:** Comparative analysis of quality of life

Quality of life variables	Interview 1	Interview 2	p*	Mean difference	Final mean difference	p**
	Mean (SEM)	Mean (SEM)		(95 % CI)	(95 % CI)	
Physical Functioning						
CRMM	28.8 (2.3)	28.2 (2.5)	0.748	−0.49 (−2.73;1.74)	−2.78 (−6.41;0.84)	0.13
Other	34.1 (3.0)	35.4 (3.1)	0.338	2.29 (−0.44;5.01)		
Physical Role Functioning						
CRMM	30.9 (1.6)	33.6 (1.5)	0.043	1.93 (−0.33;4.19)	2.89 (−0.77;6.56)	0.12
Other	36.6 (3.1)	35.9 (3.7)	0.694	−0.97 (−3.72;1.79)		
Bodily Pain						
CRMM	41.2 (3.2)	43.1 (3.1)	0.304	3.65 (1.47;5.83)	1.49 (−1.96;4.94)	0.40
Other	38.3 (2.7)	40.5 (2.8)	0.035	2.16 (−0.47;4.80)		
General Health Perceptions						
CRMM	53.7 (1.8)	50.6 (2.0)	0.158	−2.02 (−5.63;1.58)	1.91 (−4.27;8.08)	0.55
Other	43.5 (2.7)	42.8 (2.8)	0.717	−3.93 (−8.41;0.55)		
Vitality						
CRMM	47.1 (2.2)	47.8 (2.5)	0.734	1.32 (−1.68;4.31)	−2.98 (−7.72;1.76)	0.22
Other	44.1 (3.1)	49.9 (2.4)	0.004	4.30 (0.67;7.92)		
Social Role Functioning						
CRMM	45.7 (2.9)	49.2 (2.8)	0.094	4.96 (2.02;7.90)	5.81 (1.04;10.57)	0.02
Other	38.1 (2.9)	39.6 (3.0)	0.345	−0.84 (−4.43;2.74)		
Emotional Role Functioning						
CRMM	29.6 (1.5)	33.1 (1.3)	0.042	3.39 (0.80;5.99)	2.00 (−2.13;6.13)	0.34
Other	31.8 (3.0)	33.4 (3.4)	0.127	1.40 (−1.75;4.54)		
Mental Health						
CRMM	42.7 (3.3)	43.8 (3.4)	0.634	0.82 (−3.27;4.90)	−3.70 (−10.22;2.81)	0.27
Other	39.0 (3.3)	45.4 (3.1)	0.038	4.52 (−0.44;9.47)		
Physical Component Summary						
CRMM	37.9 (1.9)	37.5 (1.9)	0.729	0.03 (−1.53;1.60)	0.46 (−2.03;2.95)	0.72
Other	39.1 (2.4)	38.4 (2.5)	0.483	−0.43 (−2.32;1.47)		
Mental Component Summary						
CRMM	43.8 (2.6)	46.6 (2.7)	0.169	3.17 (0.12;6.22)	0.29 (−4.63;5.21)	0.91
Other	38.7 (2.6)	43.8 (2.4)	0.006	2.88 (−0.83;6.60)		

*Generalized Estimating Equations, Bonferroni’s corrected multiple comparisons

**Analysis of covariance, adjusted for baseline values

Table 2 describes the profile of the participants, including variables such as sex, age, site of thalidomide collection, treatment duration, and educational attainment. Of the 32 participants in the sample, 19 were female and 13 were male. Mean age was 55 years in the CRMM group and 61 years in the other facilities group (Other), with no significant difference.

**Table 2:** Demographic and treatment characteristics of the sample

Variable	CRMM	Other	*p*
	(*n* = 19) n (%)	(*n* = 13) n (%)	
Sex			0.471*
Female	10 (52.6 %)	9 (69.2 %)	
Male	9 (47.4 %)	4 (30.8 %)	
Age at diagnosis, mean (standard deviation)	55.3 (11.1)	61.3 (11.2)	0.145**
Place of residence			0.720*
Porto Alegre	7 (36.8 %)	6 (46.2 %)	
Other municipalities in Rio Grande do Sul	12 (63.2 %)	7 (53.8 %)	
Thalidomide collection site			
HCPA	19 (100 %)		
Municipal pharmacy		9 (69.2 %)	
SHD pharmacy		4 (30.8 %)	
Treatment duration in months, median (IQR)	20 (12–36)	24 (12–36)	0.248***
Educational attainment			0.351*
Primary education	9 (52.9 %)	3 (25.0 %)	
Secondary education	5 (29.4 %)	6 (50.0 %)	
Higher education	3 (17.6 %)	3 (25.0 %)	

*CRMM-HCPA* Hospital de Clínicas de Porto Alegre Referral Center for Multiple Myeloma, *IQR* interquartile range, *SHD* State Health Department

*Pearson’s Chi-square test

**Student’s t test

***Mann-Whitney’s U test

Figure 1 shows the difference in time from diagnosis to referral for transplantation between the two study groups (CRMM vs. other facilities). In each group, time from diagnosis to referral for HSCT was measured only in participants aged ≤ 65 years (*n* = 25), of whom 15 were in the CRMM group and 10 were in the other facilities group. Overall, nine patients (60 %) in the CRMM group underwent transplantation, versus five (50 %) in the other facilities group.

In this analysis, we found a significant difference (*p* = 0.036) in time elapsed between diagnosis and referral for HSCT, with patients treated at CRMM being referred for transplantation significantly sooner (median, 9 months; IQR, 8.5–14.5) than patients treated elsewhere (median, 24 months; IQR, 16–24).

**Fig. 2:**
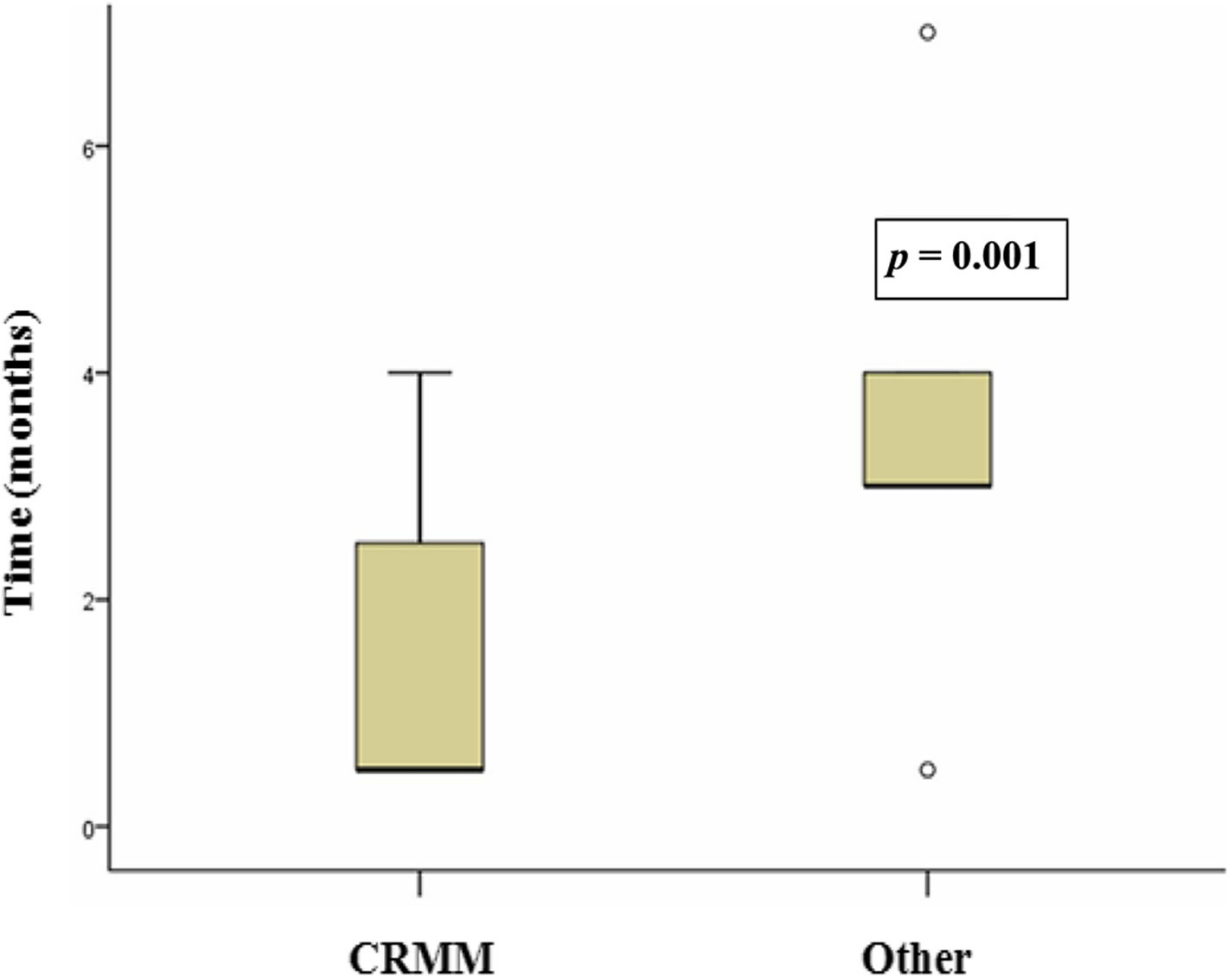
Time from diagnosis to thalidomide collection

Between-group comparison of time from diagnosis to first thalidomide dispensation (Fig. 2) again showed a significant difference (*p* < 0.001, Mann–Whitney U), with a median time to thalidomide collection of 0.5 months (IQR, 0.5–3 months) in the CRMM group versus 3 months (IQR, 3–4 months) in the other facilities group.

No significant between-group differences were found in the questionnaire items regarding treatment costs (Table 3).

**Table 3:** Comparison of treatment costs (US$)

	Interview 1	Interview 2				
Cost variable	Mean (SEM)	Mean (SEM)	p*	Mean difference (95 % CI)	Final mean difference (95 % CI)	p**
Blood tests						
CRMM	161.5 (11.8)	121.6 (16.1)	0.009	−38.51 (−64.66;-12.37)	−13.29 (−55.79;29.20)	0.54
Other	159.1 (17.0)	140.8 (19.7)	0.242	−25.22 (−58.26;7.82)		
X-rays, bone						
CRMM	27.9 (6.5)	15.6 (5.6)	0.011	−10.93 (−17.93;-3.94)	−4.87 (−16.27;6.52)	0.40
Other	19.2 (5.7)	16.9 (5.3)	0.552	−6.06 (−14.91;2.7)		
X-rays, chest						
CRMM	11.0 (3.3)	5.5 (1.9)	0.047	−6.57 (−10.40;-2.74)	−3.52 (−9.81;2.77)	0.27
Other	18.3 (3.9)	12.7 (4.1)	0.068	−3.05 (−7.91;1.81)		
Urine tests						
CRMM	92.2 (7.0)	73.6 (9.7)	0.018	−16.81 (−30.15;-3.47)	−8.66 (−31.17;13.86)	0.45
Other	59.4 (13.2)	55.6 (14.6)	0.512	−8.15 (−25.25;8.95)		
Total						
CRMM	292.6 (23.3)	216.2 (30.1)	0.003	−70.41 (−115.40;-25.41)	−24.09 (−97.32;49.15)	0.52
Other	256.0 (25.4)	226.0 (30.6)	0.268	−46.32 (−103.20;10.56)		

*Generalized Estimating Equations, Bonferroni’s corrected multiple comparisons

**Analysis of covariance, adjusted for baseline

## Discussion

Taking into account that autologous HSCT is the gold-standard treatment for MM [10, 11], any intervention that may influence this indicator or even improve the quality of life of these patients can be immensely valuable.

We found significant between-group differences in time to referral for HSCT, quality of life (specifically in the social aspect), and time between diagnosis and initiation of thalidomide therapy.

Tricot et al. found that ≤ 12 months of pharmacotherapy preceding transplantation increased event-free survival in patients with MM [12].

Therefore, the 75-day difference in referral for HSCT between patients treated at CRMM-HCPA and patients treated at other facilities may have been associated with the improved quality of life found in patients in the former group. A larger study is required to assess potential differences in survival.

### Study limitations

We initially expected to include a much greater number of participants; however, patient inclusion was fraught with difficulty, particularly because many had transportation issues that prevented them from attending questionnaire administration visits at the study site and because of incorrect data in the SHD registry, which precluded contact with many potential participants. In addition, the small sample size and limited evaluation time did not allow us to draw any conclusions as to the potential impact on patient survival.

Furthermore, the fact that questionnaire items asked participants to recall events occurring in the “last 6 months” may have been a hindrance, as we believe reliable recall this far back may be quite difficult for certain items. Nevertheless, we did not conduct a formal assessment of whether this factor could introduce bias or interfere with results, as a similar 6-month period is used in other, validated questionnaires, such as the SF-36 v2.

We found that few studies have assessed the efficacy or cost-effectiveness of SUS services in Brazil, which limited the methodological framework available to us. HCPA is the only facility in the state of Rio Grande do Sul that provides a RC for MM. The presence of other such centers in the state, at other facilities where patients collect SHD-provided medicines, would have enabled a multicenter analysis and increased the sample of participants, which would have improved the quality of the results obtained.

The findings of this study may be useful to other Brazilian states and even to other countries.

## Conclusions

The RC treatment strategy seems to be more efficient than the comparator group, as it facilitated access to medicines and enabled a significant reduction in time elapsed between diagnosis of MM and referral for HSCT.

Furthermore, patients treated in the RC setting demonstrated greater ease in performing social activities – such as visiting family, friends, and neighbors – with less interference from physical and emotional problems as compared with patients treated at the other facilities.
